# Prostruc: an open-source tool for 3D structure prediction using homology modeling

**DOI:** 10.3389/fchem.2024.1509407

**Published:** 2024-11-29

**Authors:** Shivani V. Pawar, Wilson Sena Kwaku Banini, Musa Muhammad Shamsuddeen, Toheeb A. Jumah, Nigel N. O. Dolling, Abdulwasiu Tiamiyu, Olaitan I. Awe

**Affiliations:** ^1^ Department of Biotechnology and Bioinformatics, Deogiri College, Auranagabad, Maharashtra, India; ^2^ Department of Theoretical and Applied Biology, College of Science, Kwame Nkrumah University of Science and Technology, Kumasi, Ghana; ^3^ Department of Public Health, Faculty of Health Sciences, National Open University of Nigeria, Abuja, Nigeria; ^4^ School of Collective Intelligence, University Mohammed VI Polytechnic, Rabat, Morocco; ^5^ Department of Parasitology, Noguchi Memorial Institute for Medical Research, University of Ghana, Accra, Ghana; ^6^ African Society for Bioinformatics and Computational Biology, Cape Town, South Africa

**Keywords:** homology modeling, protein structure prediction, biopython, BLAST, ProMod3, open-source software

## Abstract

**Introduction:**

Homology modeling is a widely used computational technique for predicting the three-dimensional (3D) structures of proteins based on known templates,evolutionary relationships to provide structural insights critical for understanding protein function, interactions, and potential therapeutic targets. However, existing tools often require significant expertise and computational resources, presenting a barrier for many researchers.

**Methods:**

Prostruc is a Python-based homology modeling tool designed to simplify protein structure prediction through an intuitive, automated pipeline. Integrating Biopython for sequence alignment, BLAST for template identification, and ProMod3 for structure generation, Prostruc streamlines complex workflows into a user-friendly interface. The tool enables researchers to input protein sequences, identify homologous templates from databases such as the Protein Data Bank (PDB), and generate high-quality 3D structures with minimal computational expertise. Prostruc implements a two-stage vSquarealidation process: first, it uses TM-align for structural comparison, assessing Root Mean Deviations (RMSD) and TM scores against reference models. Second, it evaluates model quality via QMEANDisCo to ensure high accuracy.

**Results:**

The top five models are selected based on these metrics and provided to the user. Prostruc stands out by offering scalability, flexibility, and ease of use. It is accessible via a cloud-based web interface or as a Python package for local use, ensuring adaptability across research environments. Benchmarking against existing tools like SWISS-MODEL,I-TASSER and Phyre2 demonstrates Prostruc's competitive performance in terms of structural accuracy and job runtime, while its open-source nature encourages community-driven innovation.

**Discussion:**

Prostruc is positioned as a significant advancement in homology modeling, making high-quality protein structure prediction more accessible to the scientific community.

## 1 Introduction

Proteins, which are some of the most common and intricate macromolecules in living organisms, have garnered considerable interest in biological research. Proteins differ primarily based on their amino acid sequences, which lead them to possess distinct spatial shapes and structures, resulting in different biological functions within cells. Although our knowledge of structural chemistry has greatly improved over the years, our comprehension of the mechanisms by which proteins get their distinctive three-dimensional conformations from their linear sequences remains constrained. X-ray crystallography and NMR spectroscopy are the two primary experimental methods for elucidating protein structures. Nonetheless, both approaches are laborious and time-consuming, and they possess technological constraints contingent upon the protein targets. Consequently, in recent decades, researchers have endeavored to devise very sophisticated computational approaches for predicting enhanced protein structures (Deng et al., 2018).

Homology modeling, also termed comparative modeling or template-based structural prediction, is a programmatic technique implemented using a variety of programming languages for predicting the three-dimensional structure of a protein by leveraging its sequence similarity to a single or multiple established existing structures (Martí-Renom et al., 2000). This method leverages the evolutionary relationship between proteins to infer the structure of a target protein from its homologous counterparts. Accurate modeling of protein structures is crucial in several domains, such as protein engineering and drug discovery (Chothia and Lesk, 1986).

A fundamental observation is that similar sequences from the same evolutionary family often adopt similar protein structures, forming the basis of homology modeling. This method remains the most accurate way to predict protein structures by using homologous structures in the PDB/mmcif format as templates (Deng et al., 2018). With the rapid expansion of the PDB database, an increasing proportion of target proteins can be predicted through homology modeling. Protein targets are important in the development of therapies for complex diseases (Ogbodo et al., 2023). Even when no structure with obvious sequence similarity to the target protein is found in the PDB, it is still possible to identify proteins with structural similarity to the target protein (Berman et al., 2000). This method, known as threading or fold recognition, matches the target sequence to homologous and distant-homologous structures using specific algorithms and uses the best matches as structural templates. The underlying premise for threading is that protein structure is highly conserved through evolution, and the number of unique structural folds is limited in nature (Peng and Xu, 2009).

Sequencing has played a significant role in the advancement of computational biology. For instance, there have been research efforts which aimed to model the evolution of viral pathogens like SARS-CoV-2 using genomic sequence data (Mwanga et al., 2023; Awe et al., 2023), HIV-1 evolution in sub-Saharan Africa (Obura et al., 2022), biomarker discovery (Chikwambi et al., 2023; Nyamari et al., 2023; El Abed et al., 2023; Ben Aribi et al., 2024; Alaya et al., 2024), malaria/COVID-19 biomarker discovery (Nzungize et al., 2022), analysis of RNA-seq and ChIP-seq data (Ather et al., 2018), and Ebola Virus comparative genomics (Oluwagbemi and Awe, 2018). Genomics and bioinformatics have also been reported to be playing a key role in newborn screening (Wesonga and Awe, 2022) and in agriculture. There have been computational methods to define gene families and their expression in legumes (Jose et al., 2019). Homology modeling, which relies on sequence comparison, is different from the *ab initio* technique, which seeks to construct structures solely on fundamental physical principles without dependence on any previously resolved structures. However, the scarcity of perfect, effective *ab initio* procedures, primarily due to the folding process of a one-dimensional amino acid sequence into a three-dimensional protein structure, necessitates the resolution of numerous protein structures using comparative modeling (Deng et al., 2018; Yang et al., 2022).

Various homology modeling tools have been designed over the years, and have significantly contributed to our understanding of protein structures. Existing homology modeling tools, such as SWISS-MODEL, MODELLER, and Phyre2 have been widely used and have provided an avenue for various research in the bioinformatics world (Schwede et al., 2003; Arnold et al., 2006). However, most of these tools often require substantial computational resources, specialized software installations, or advanced user expertise, making them less accessible to researchers and practitioners who may not have a computational background. Moreover, the integration of these tools into a seamless and user-friendly web interface remains a challenge (Webb and Sali, 2016).

To address these limitations, newer homology modeling tools are continuously being produced, mostly web-based, which are refined in various aspects to enhance usage, accessibility, and provide robust computational capabilities. Such a tool should enable researchers from diverse backgrounds to perform homology modeling without the need for extensive computational infrastructure or expertise (Kelley et al., 2015). Additionally, it should provide an intuitive interface that guides users through the modeling process, from sequence input to structure visualization and validation (Yang et al., 2015). Currently, most homology modeling is offered as a web-based service, however, an open-source python package would serve as a significant milestone in the field, which is one of the major aims of this project.

## 2 Methods

### 2.1 Release options and workflow

**FIGURE 1:**
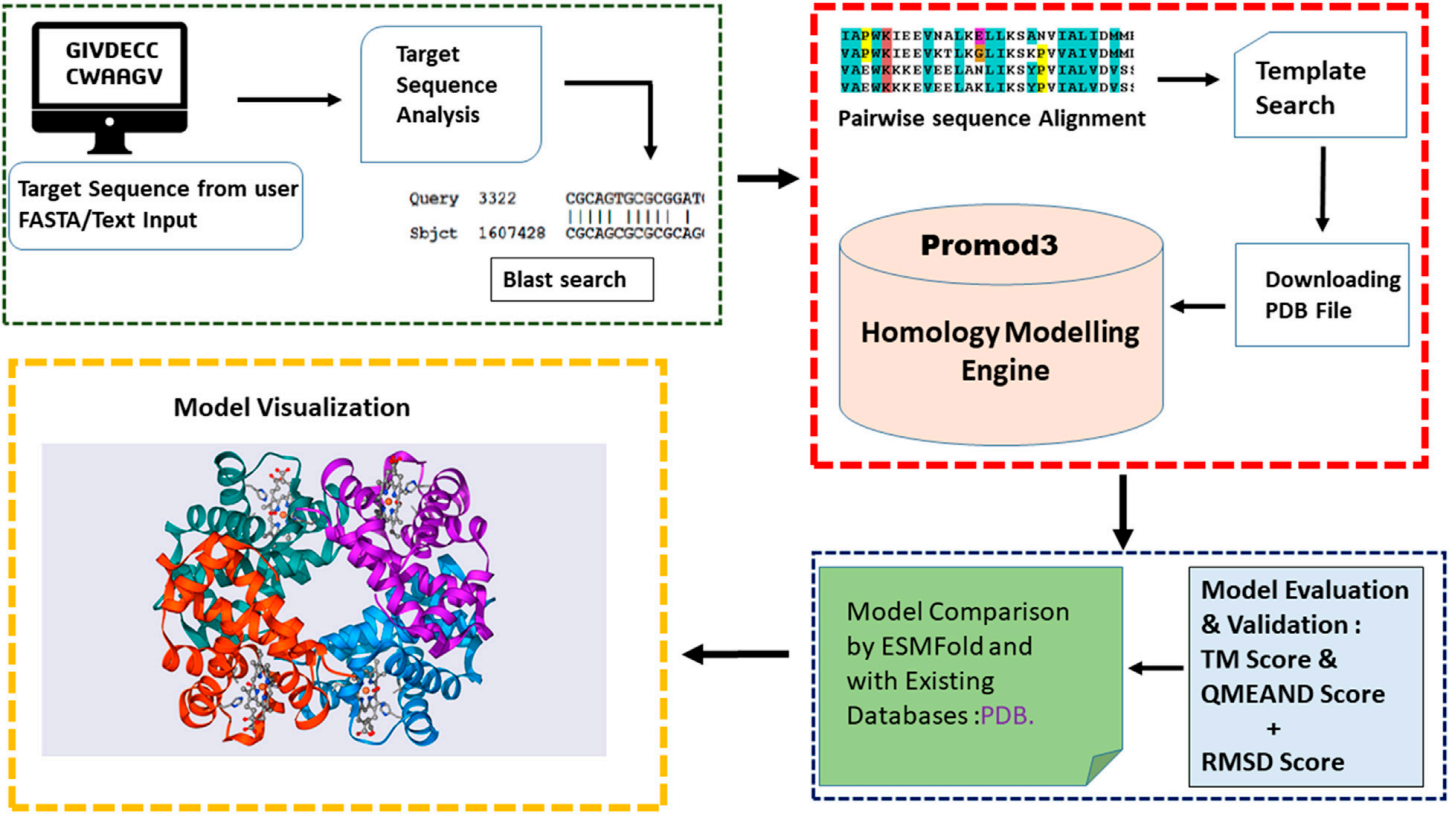
Prostruc workflow.

Prostruc was primarily developed in Python, leveraging a variety of libraries and third-party tools to facilitate robust protein structure prediction (Figure 1). As has already been established, one of the main design decisions was to create a tool that homology modeling novices can use with ease. Hence, a cloud based server has been made available to host the service. For more technical or advanced users who may want to extend the tools functionality or improve their automation pipelines, a python package has also been published on PyPi.org.

#### 2.1.1 Web environment

The web version of the tool operates on a cloud-based server running the Linux operating system, specifically Ubuntu 20.04 LTS. The web interface was crafted using Streamlit (v1.24.0), which also provides the underlying web server infrastructure as it is bundled with the package. This setup allows for a seamless user experience with an intuitive and responsive interface. To efficiently manage multiple user requests and execute tasks independently and concurrently, Celery (v5.3.1) was integrated into the environment. Celery ensures that the various tasks associated with structure prediction are handled in parallel, improving overall performance and scalability. The core modeling engine, ProMod3 (v3.2.1), operates within a Docker container, ensuring a consistent and isolated environment for model generation. This containerized approach enhances reproducibility and simplifies deployment across different systems. The Prostruc environment thus comprises three main services running in the background: Streamlit for the web interface, Celery for task management, and Docker for the ProMod3 modeling engine. Together, these components create a cohesive and scalable environment for protein structure prediction.

#### 2.1.2 Prostruc python package

Relative to the web tool, the python package requires only the docker service to be running in the background. The other services were not added to the package since no job management will be required. Similar to the web tool, The three major third-party libraries that the package depends on are biopython, docker, and reportlab.

#### 2.1.3 Workflow

### 2.2 Sequence retrieval and validation

Protein sequences can be provided by users through the tool’s web interface or the python package. Upon submission, each sequence underwent initial validation using Biopython’s SeqIO (Cock et al., 2009) module to ensure correct format and completeness. To maintain computational efficiency and accommodate underlying limitations within BioPython, Prostruc enforces a maximum sequence length of 400 amino acids. Sequences exceeding this length are rejected, and users are prompted to submit shorter sequences.

### 2.3 Homologous template search

The identification of potential templates is achieved using the Basic Local Alignment Search Tool (BLAST) against the Protein Data Bank (PDB) (Berman et al., 2000). Using NCBI’s blastp program, templates were selected based on sequence similarity and structural relevance. Template candidates were filtered based on their e-value and identity score, with stringent criteria set to retain only highly similar templates. Specifically, a minimum identity threshold of 30% and an e-value cutoff of 0.01 were used to ensure the selection of reliable templates for subsequent modeling.

### 2.4 Sequence alignment

Pairwise sequence alignment was performed using Biopython’s Pairwise module, saving the result of the alignment as a fasta file in a designated directory. While multiple sequence alignment is often employed in other homology modeling pipelines, employing tools such as MAFFT (Katoh et al., 2002; Kuraku et al., 2013), MUSCLE (Edgar, 2004), T-Coffee (Notredame et al., 2000) and Clustal-Omega (Sievers et al., 2011), our approach focused on pairwise alignment due to compatibility with ProMod3. Initial tests revealed that ProMod3 does not accept alignment files containing more than two sequences, making pairwise alignment the optimal choice for this workflow.

### 2.5 Building the model

The alignment files, along with the selected homologous templates, were input into the ProMod3 (Studer et al., 2021) modeling engine, which runs within a Docker container. Successfully generated models were automatically stored in a designated directory, pending further evaluation and validation. This integrated approach, with ProMod3 at the core of the structural building process, provided a robust framework for high-quality protein structure prediction.

### 2.6 Model evaluation and validation

To ensure the accuracy and reliability of the predicted models, Prostruc implements a comprehensive two-stage validation process. This rigorous approach is designed to filter out suboptimal models and retain only the most accurate predictions. The details about the validation process is as follows:

Stage 1: Structural Comparison using TMAlign: The first stage of validation focuses on structural comparison, where each predicted model is evaluated against a reference validation model generated by ESMFold. TMAlign, a widely recognized tool in structural biology, is employed for this purpose. TMAlign calculates two key metrics:
  • Root Mean Square Deviations (RMSD): RMSD measures the average distance between corresponding atoms of the predicted and reference structures. It is a crucial metric for assessing the overall geometric accuracy of the model. Lower RMSD values indicate better alignment with the reference structure.
  • TM Score (Template Modeling Score): The TM score assesses the topological similarity between the predicted model and the reference structure. Unlike RMSD, which can be sensitive to outliers, the TM score provides a normalized measure of similarity that is less dependent on model size. A TM score ranges from 0 to 1, with scores closer to 1 indicating higher structural accuracy. In Prostruc, models with TM scores below 0.5 are considered structurally inadequate and are discarded.
Stage 2: Quality Assessment using QMEANDisCo: The models that pass the initial structural comparison undergo further validation in the second stage, where the focus shifts to model quality. This stage utilizes QMEANDisCo, a quality assessment tool provided by SWISS-MODEL.

QMEANDisCo evaluates the model by analyzing various statistical potentials derived from the model’s geometry and comparing them with high-resolution experimental structures. This method combines distance constraints with other factors such as torsion angles and solvent accessibility to provide a comprehensive quality score. The QMEAN score is a composite metric where values closer to 1 indicate higher confidence in the model’s structural accuracy. In Prostruc, models with a QMEAN score below 0.6 are rejected to maintain a high standard of prediction accuracy.

### 2.7 Final model selection

After both stages of validation, the top five models with the highest TM and QMEAN scores are selected. These models are then delivered to the user via email, ensuring that only the most reliable and structurally accurate predictions are provided.

### 2.8 Error handling and job management

To enhance the robustness of the Prostruc pipeline, extensive measures were taken to handle potential errors and failed jobs gracefully. Whether due to input issues, computational errors, or external factors, any failure within the pipeline triggers an automated response system that notifies the user via email and updates the job status on their dashboard. The Python package also alerts the user with the appropriate message in the event that an error occurs or a job fails to complete. This ensures transparency and allows users to take corrective action as needed.

### 2.9 Prostruc pipeline validation and optimization

The validation of the Prostruc pipeline is conducted by benchmarking its performance against established protein structure modeling tools, including SWISS-MODEL, MODELLER, and PRIMO. The evaluation focuses on the accuracy and quality of the 3D models generated by these tools in comparison to those produced by Prostruc. Key metrics used in the analysis include:1. QMEANDisCo 2. Root Mean Square Deviations (RMSD) 3. TM Scores.

Other existing tools were also compared against each other to determine their individual strengths and unique features.

Through these comparative analyses, Prostruc’s performance is continuously validated and optimized, ensuring that it remains competitive with other leading tools in the field of protein structure prediction.

## 3 Results

### 3.1 Prostruc python package

The Prostruc python package was designed as a cross-platform tool that is intended to work efficiently in all major environments. Tests were carried out using Google Colab, Jupyter Notebook, Conda, and Pycharm, the package works well on all these platforms. The user must ensure docker is installed and running in the background. registry.scicore.unibas.ch/schwede/promod3:latest is the promod3 docker image Prostruc depends on. edraizen/tmalign:latest is the docker image for TM-Align, which is used to calculate the TM-Score and RMSD score. It is recommended that users add these images to their local docker service before installing Prostruc. Prostruc can however automatically install these images in case the user fails to do so. The following shows how to easily install the package and get started:

• To install docker: *sudo apt install docker. io*
• To start docker service: *sudo systemctl start docker*
• To pull the images: *sudo docker pull* registry.scicore.unibas.ch/schwede/promod3:latest *sudo docker pull* edraizen/tmalign:latest
• To install Prostruc: *pip install prostruc*
• To run job: *prostruc—sequence “AAAA” —job_name “descriptive_job_name” —email “user@example.com”*

Users have to ensure that they have a stable internet connection, especially during the first time the tool is being used.

### 3.2 Prostruc web user interface and pipeline

**FIGURE 2:**
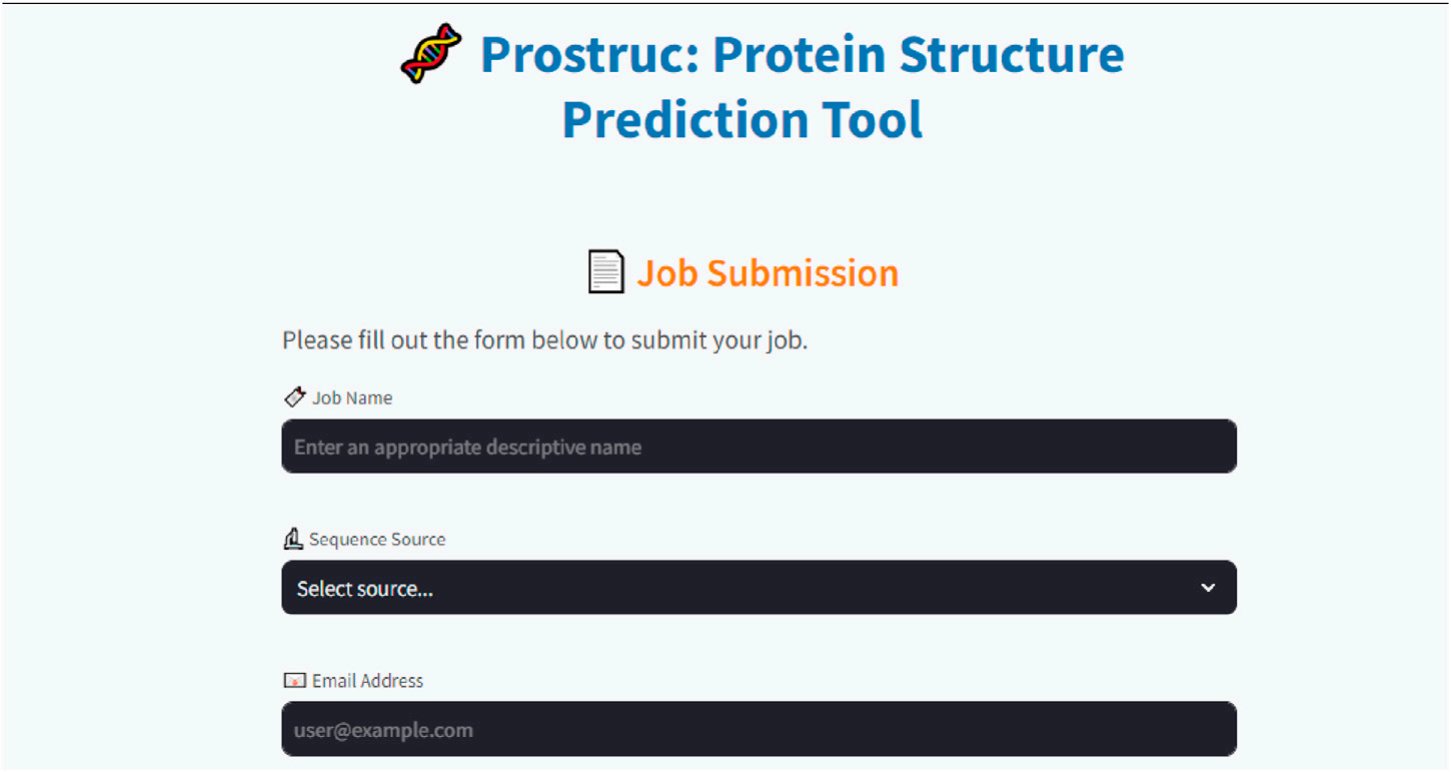
Job submission page.

The Prostruc user interface (Figure 2) was designed using Streamlit, chosen for its simplicity and robustness. Streamlit’s capabilities allowed the development team to concentrate on the core functionality of the pipeline without being sidetracked by time-consuming UI design challenges. The UI code is primarily written in Python, with minimal HTML and CSS integrated where necessary to enhance the interface. The side panel features two main sections: the Job Submission page and the Status page. Users can effortlessly input a unique job name, define their sequence source, and an email address before submitting their jobs.

The interface shows a complete overview of the modeling job, allowing users to simply type-in their sequence source or import its FASTA file and instantly begin the modeling process. Prostruc by default is set to run using the preset parameters at each modeling stage which provides results within a shorter period of time. However, the user has the liberty to modify the parameters for template identification, sequence alignment and modeling which could invariably increase the job’s running time. Template identification is primarily done using the BLAST suite (Altschul et al., 1997).

**FIGURE 3:**
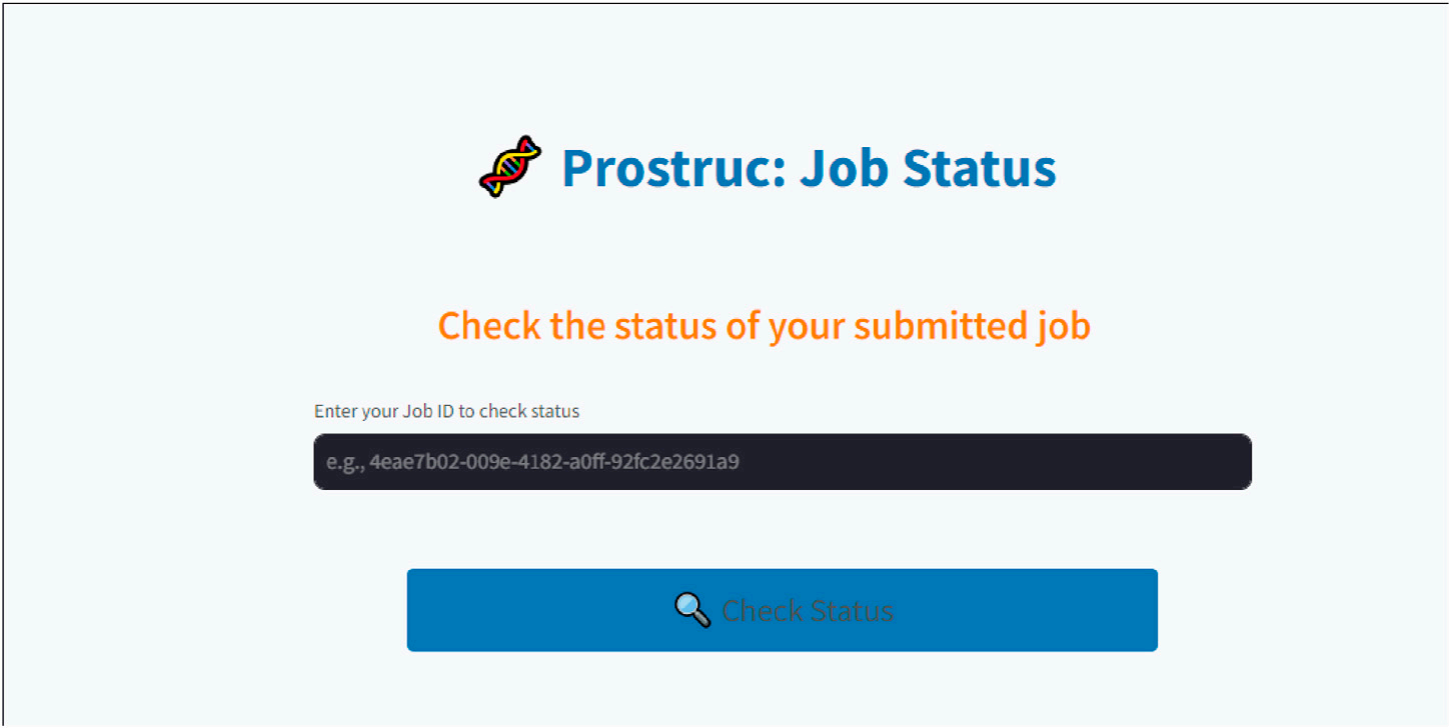
Status page.

The following are the major parts of the web UI and pipeline:

• Job Dashboard: The job dashboard (Figure 3) corresponds to the user interface that is shown to the user upon sequence submission. Users are redirected to their personalized dashboard, which provides real-time updates on the status of their job with the display user interface “Prostruc: Job Status.” The dashboard is designed to give users clear visibility into the current stage of the pipeline their job is in. Whether the job is pending, processing, or completed, users are continuously informed about the specific operation being performed.
• Job Processing: The only inputs required from users are the job name, sequence source, and email address (Figure 2). Once a job is submitted, it is added to a job queue managed by Celery. If the server is currently occupied with other tasks, the new job is placed in the pending queue. As soon as resources become available, the job is moved to the processing stage, where it progresses through the various stages of the pipeline. Depending on factors like sequence length, server load, and network conditions, job completion times may vary.
• Job Management: For effective job management, a unique ID is generated for each job and sent to the user via email. Users can return to the Prostruc website at any time and use their job ID to check the status of their job. Celery utilizes this job ID to manage jobs independently, ensuring smooth task handling. Upon job completion, the modeled structures are emailed to the user. If an issue arises during processing, the user is promptly notified with detailed feedback and suggestions for resolving the problem. Additionally, users have the option to contact the Prostruc support team via email for further assistance.

### 3.3 Structure prediction and performance analysis

The efficiency and accuracy of the Prostruc pipeline can be influenced by various factors, including the characteristics of the target sequence submitted as the sequence source. To assess its performance, the pipeline was tested with different protein sequences. Among the stages, model generation using ProMod3 and validation using QMEANDisCO were identified as the most time-consuming processes. QMEANDisCO relies on SWISS-MODEL’s server for validation, which contributes to the extended runtime.

One of the test sequences (for the purpose of this paper, it will be referred to us TestSeq) used in the evaluation was derived from an alpha-amylase (Uniprot:Q98TR6; The UniProt Consortium, 2023) enzyme, a protein involved in the hydrolysis of starch into sugars:

FEWRWADIAAECERFLGPNGFGGVQISPPNDHIVLNNPWRPWWQRYQPIGYNLCSRSGSENELRDMITRCNNVGVNIYVDAVINHMCGAGGGEGTHSSCGSWFSAGRRDFPTVPYSHLDFNDNKCRTGSGDIENYGDSNQVRDCRLVGLLDLALEKEYVRGKVVDFMNKLIDMGVAGFRVDACKHMWP

**FIGURE 4:**
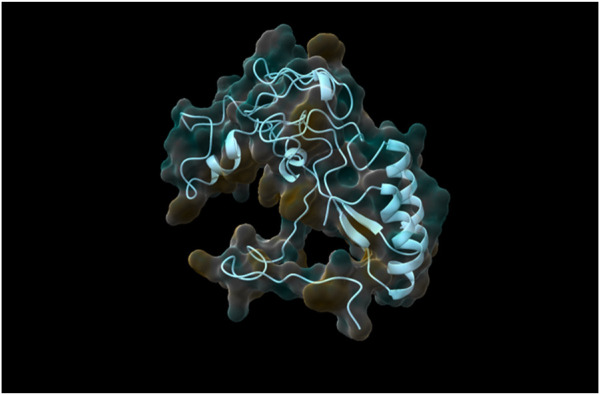
Generated Model 1.

**FIGURE 5:**
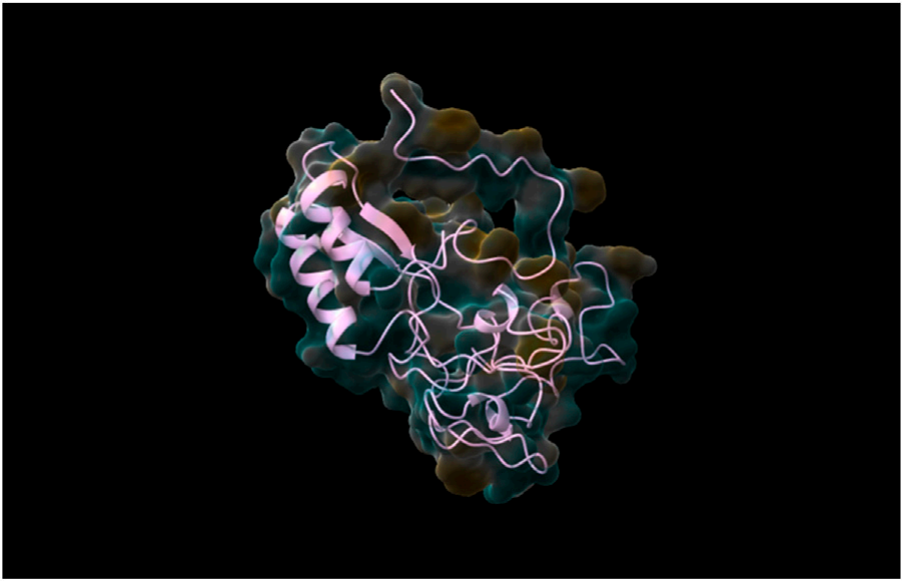
Generated Model 2.

**FIGURE 6:**
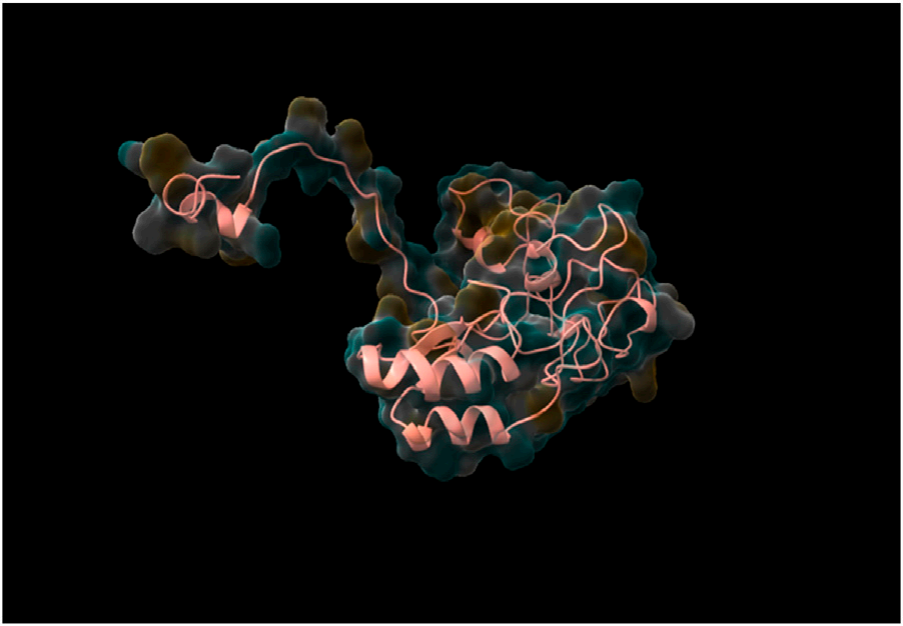
Generated Model 3.

Following the complete run through the pipeline using the web tool, the top five models were identified and returned to the user via email. The best-performing model, determined through rigorous validation, achieved a QMEAN score of 0.87, indicating a high-quality structure prediction. As was already stated, structure validation using QMEAN took most of the time. TestSeq was also run through SWISS-MODEL and the output models were compared to Prostruc’s. Figures 4–6 are the top 3 best-performing models.

### 3.4 Prostruc performance compared to other tools

In evaluating Prostruc against established homology modeling tools, we considered not only the quality of the predicted models but also key factors such as job runtime and user experience. Prostruc was specifically compared to SWISS-MODEL, one of the most widely recognized tools in the field. The results of this comparison are summarized in Table 1. Comparisons between the major homology modeling tools are summarized in Table 2. We also compared performance metrics of Prostruc with other widely used tools, as shown in Table 3. The following are the parameters that were considered when comparing Prostruc with SWISS-MODEL:

1. Job Runtime:
  ○ Usefulness: Very relevant, as faster runtimes are often preferred, especially in high-throughput scenarios.
2. Number of Output Models:
  ○ Usefulness: Useful, as some users may require multiple models to assess structural variability.
3. Model Quality:
  ○ Usefulness: Critical, as the accuracy and reliability of the predicted models are the most important aspects.
4. Novice Friendly (User-Friendliness):
  ○ Usefulness: Important for users with varying levels of expertise. A more intuitive interface can lower the barrier to entry.
5. Open Source:
  ○ Usefulness: Relevant, as open-source tools often allow for more flexibility, community support, and customization.
6. Scalability:
  ○ Description: How well does the tool handle large-scale or batch processing? Can it be easily integrated into automated workflows?
7. Template Library Coverage:
  ○ Description: The breadth and depth of the template library used for homology modeling.
8. Customization and Flexibility:
  ○ Description: The ability to tweak parameters, add custom scripts, or integrate additional tools. Useful for advanced users who require more control over the modeling process.

**TABLE 1:** Comparison between prostruc and SWISS-MODEL.

Parameters	SWISS-MODEL	Prostruc
Job runtime	Generally shorter due to optimized backend and efficient template use.	Longer, especially with detailed structural validation (e.g., ProMod3 and QMEANDisCO).
Number of output Models	2	5
Novice Friendly (User-friendly)	Yes	Yes
Open Source	No: Proprietary, limiting customization	Yes: Open-source, allowing for extensive modification and integration.
Scalability	Limited - as SWISS-MODEL is a web-based service with fixed resources and does not allow for extensive customization.	High - Prostruc can be scaled by deploying it on cloud servers, and additional tools or models can be integrated.
Template library coverage	Extensive, SWISS-MODEL uses a large library of templates derived from the PDB and other sources.	Flexible, Prostruc allows users to explore various databases for template searches, potentially offering broader coverage.

**TABLE 2:** Comparing various template-based protein structure modeling (homology modeling) tools.

Tool	Description	Web server	Model optimization method	Model validation method	Template database source	Modeling methodology	Installable package/Downloadable program
Prostruc	A Python-based Tool for Homology Modeling and 3D Structure Prediction using BLAST search for sequence alignment.	Yes	Energy minimization	QMEANDisCO, TM-score, and RMSD.	PDB	Homology modeling using ProMod3 to predict protein structure after multiple pairwise alignments between the templates and the sequence source submitted by users. The structures generated by ProMod3 are also compared to a single structure generated by ESMFold directly from the sequence to validate the highest quality protein structure from ProMod3.	Yes
SWISS-MODEL (Waterhouse et al., 2018)	A fully automated protein structure server that uses BLAST or HHsearch for sequence alignment search.	Yes	Energy minimization	ProSA (GMQE, QSQE) and ProQ (QMEAN, QMEANDisCo, QMEANBrane)	UniProt and PDB	Homology modeling using ProMod3.	No
EasyModel (Arab and Dantism, 2023)	A protein structure prediction server for structural prediction using comparative protein modeling.	Yes	MODELLER optimization parameter (VTFM with CG, MD&SA)	DOPE score	PDB	Homology modeling using MODELLER.	Yes
Phyre2 (Kelley et al., 2015)	A sophisticated modeling tool that uses PSI-BLAST and HHpred for structure similarities search.	Yes	Jensen-Shannon Divergence, CSA, fpocket2, SuSPect, detecting sequence features from Conserved Domain Database (CDD), and interface detection using ProtinDb and PI-site.	ProQ2 and Molprobity	PDB and Uniprot	Homology modeling using MODELLER, *ab initio* folding simulation for area with no homology detection using Poing algorithm, and transmembrane helices prediction using Memsat-svm.	No
RaptorX (Källberg et al., 2012)	A valuable tool for predicting structures of novel proteins with limited sequence similarity to known structures.	Yes	DISOPRED and domain parsing.	TM-score, probabilistic-consistency algorithm and entropy–dependent scoring function.	Pfam and PDB	Homology modeling using MODELLER, secondary structure prediction pipeline using CNF model, protein threading to improve sequence-template alignment, and T-Coffee algorithm to generate quality alignments.	Yes
I-TASSER (Roy et al., 2010)	A multi server structural and functional prediction tool based on the inter-relationship between protein sequence to its structural model and then to its functional attributes. It uses PSI-BLAST to identify similar structure motifs.	yes	TM-align, SPICKER, and REMO.	Z-score, GO-score,TM-score, RMSD, and C-score.	PDB	Model building using multiple-threading alignments by LOMETS (Local Meta-Threading Server, version 3) and iterative threading assembly refinement (I-TASSER). Secondary structure is predicted using PSIPRED, and the fragment assembly is conducted using a modified replica-exchange Monte Carlo simulation technique.	Yes
Robetta (Raman et al., 2009)	An integrated protein structure prediction server that uses multiple sequence search for quality homologs. Profile-to-profile alignment using COMPASS and PROCAIN, and BLAST and PSI-BLAST/HHpred for target-template similarity search.	Yes	PROMALS, loop-modeling, and Monte Carlo trajectory.	minimum-RMSD, LiveBench contact scores, GDT-TS Z-score, GDT-TS, GDT-HA.	PDB and NCBI non-redundant protein database.	Construction of multiple sequence alignment using PROMALS3D, building of hybrid templates using DALI, and homology modeling and *de novo* prediction using Rosetta.	No
GPCRautomodel (Launay et al., 2012)	A protein structure prediction tool for GPCR proteins based on multiple sequence similarity from BLAST	Yes	In-house threadings	RMSD	PDBTM database	Threading of remote homologs using FROST. Prediction of the model using MODELLER based on the template obtained from the FROST alignment.	No
ModWeb (Pieper et al., 2011)	A web server protein structure prediction tool that builds protein structure through an automated modeling pipeline called ModPipe. It uses BLAST to search for sequence similarities.	Yes	Self -organizing map	GA341 score, MPQS score, Z-DOPE score, TSVMod score	UniProtKB, PDB	The ModPipe modeling pipeline built its protein structure based on Homology modeling using MODELLER. It also uses sequence to sequence, sequence to profile and profile to profile for building the alignment between target and template.	No
PROTEUS2 (Montgomerie et al., 2008)	A multifaceted web-based server that also includes predicting protein 3D structure using homology modeling. It also uses BLAST for template sequence search.	Yes	Energy minimization using GAfolder	Average hydrogen bond energies, all-atom RMSD, backbone RMSD, threading energies, and bump scores.	PDB	Homology modeling using HOMODELLER.	No
HHpred (Hildebrand et al., 2009)	A web based server for the prediction of protein structure developed by Max Planck Institute for Biology. It uses HHsearch for sequence similarity templates.	Yes	MAC algorithm, domain parsing procedures, Viterbi algorithm, and Stuttgart Neural Network Simulator.	HHsearch score, Gonnet matrix score, sum_probs scores and TM-score.	PDB, HMM database, and NCBI non-redundant protein database.	The hidden Markov model is used to generate template homologs using the hhmake. Homologous templates are distinguished from nonhomologous templates using the HHsearch ranks database. An homology model is built for each target-template alignment using Modeller. The neural network then predicts a TM-score for each model that is built in correspondence to the target template to rank closely similar structures.	Yes
Multiple Mapping Method with Multiple Templates (M4T) (Fernandez-Fuentes et al., 2007)	A protein structure prediction tool that uses multiple mapping of multiple templates generated by PSI-BLAST sequence similarities search.	Yes	BlastProfiler	LGA_S score, DOPE score, and PROSA2003 score.	PDB and NCBI non-redundant protein database.	Multiple templates are obtained from PSI-BLAST search using three iterations. The target-template sequence is further search against the NCBI nr database to build the multiple mapping over five iterations. The Selected template from the above framework is then used to build the protein structure model using Modeller.	No
PSIPRED (Jones, 1999)	A protein structure prediction tool that utilizes a two-stage neural network. It also uses PSI-BLAST for generating sequence similarities.	Yes	SEG program and position-specific scoring matrix (PSSM).	Q_3_ scores and Sov_3_ scores.	PDB	The PSSM of the protein homologs is generated and fed into the first stage neural network. The initial predictions from the first neural network are fed into the second neural network which refine the predictions by considering correlations between residues. The model is further used to predict protein structure.	Yes
ESyPred3D (Lambert et al., 2002)	An Homology modeling tool that uses PSI-BLAST for sequence similarities search.	Yes	Spatial and geometric restraints and molecular dynamic annealing.	AL0 score and GDT TS score.	PDB and NCBI non-redundant protein database	Five different alignment programs such as ClustalW and Match-Box are used to build the multiple alignment of the template search. The output is stored in the database and redundant information is discarded. The final output is used to build the 3D structure using Modeller.	No

**TABLE 3:** Performance comparison of prostruct with other homology modeling tools.

Tool name	Execution time (minutes)	RMSD (Å)	TM score	QMEANDisCo	QMEAN
Prostruc	∼10–40	0.5	0.62	0.42	0.87
Swiss-Model	∼15	1.0	0.75	0.68	0.79
I-Tasser	>60	1.2	0.78	0.72	0.80
Phyre2	∼37–42	1.5	0.70	0.65	0.70
EasyModel	∼15–120	1.2	0.65	0.58	0.75
PSIPRED	∼15–30	1.5	0.68	0.65	N/A
PROTEUS2	45–120	1.1	0.73	0.65	0.75
HHpred	2–50	2.5	0.60	0.50	N/A

### 3.5 Source code, scripts and docker images

All source code, including third-party scripts and tools used in the development process, is available on the Prostruc GitHub repository. The following are the links to the ProMod3 and TM-Align docker images:

• ProMod3: registry.scicore.unibas.ch/schwede/promod3:latest
• TM-Align: edraizen/tmalign:latest

This transparency allows users, researchers, and developers to explore the inner workings of Prostruc and contribute to its ongoing development.

## 4 Discussion

The primary objective of this study was to develop Prostruc, as an open-source homology modeling tool designed to maximize customization and scalability. Many existing tools, such as MODELLER, are proprietary and come with restrictive licenses, limiting access for many researchers, particularly those in academic or resource-constrained environments. These proprietary tools also restrict customization, impeding rapid advancements by constraining users’ ability to innovate and refine methodologies.

Prostruc addresses these challenges by providing an open-source, Python-based platform that enhances accessibility and flexibility. Python’s popularity and robust support for scientific computing made it an ideal choice for this tool. Prostruc’s open-source nature not only allows for extensive customization but also supports community-driven enhancements, making it a valuable resource for advancing homology modeling.

Unlike other tools out there, Prostruc provides both a web interface that users can interact with and a python package that is aimed at allowing researchers to build models on their machines locally. This will make it possible for researchers and developers to easily integrate the tool’s features into their workflows.

The performance comparison between Prostruc, SWISS-MODEL and Other Tools, summarized in Table 1, highlights several key differences. SWISS-MODEL demonstrated a shorter runtime, which is advantageous for rapid predictions. In contrast, Prostruc’s reliance on the QMEANDisCo validation service, provided by SWISS-MODEL, results in a longer job runtime. However, Prostruc excels in user-friendliness with its intuitive interface, making it accessible even to less experienced users. SWISS-MODEL offers extensive configuration options, which, while beneficial for advanced users, may introduce complexity.

Overall, while SWISS-MODEL and the other tools produced models with slightly better stereochemistry and side-chain conformations, Prostruc offers competitive performance, particularly in minimizing Ramachandran outliers and maintaining bond geometry. Its ability to generate multiple models and its open-source nature makes it a strong alternative for detailed structural analysis and exploratory research. The open-source nature and scalability of Prostruc suggest its potential for adaptation in large-scale proteome studies and integration with machine learning models, paving the way for more nuanced and comprehensive structure predictions. This flexibility could be particularly valuable in fields such as drug discovery, where structural diversity is critical.

Among the numerous challenges faced during the development of Prostruc, the most significant was access to structural validation tools. This issue is a major contributor to Prostruc’s longer runtime. Comprehensive validation tools are crucial for ensuring the accuracy and reliability of predicted structures but often require substantial computational resources and time. Addressing this challenge involves balancing the need for thorough validation with practical constraints of runtime and resource usage. As Prostruct continues to evolve, optimizing these aspects will be key to improving its efficiency while maintaining high standards of structural analysis.

## 5 Conclusion and next steps

The Prostruc pipeline provides a flexible and effective method for homology modeling that can easily be integrated into unique workflows where necessary. Prostruc simplifies the difficult process of predicting protein structures so that researchers with varying levels of computational experience can use it, enabling high-throughput structural biology research. In addition to making the investigation of protein interactions and functions easier, this technology advances the science by offering a versatile, user-friendly platform for both novices and more advanced users. One of the major next steps is to dockerize most of the open-source validation tools as this will improve local job processing using the python package. That being said, Prostruc is a freely accessible resource that has the potential to greatly improve protein structure prediction’s accessibility and efficiency, hence furthering our understanding of protein biology.

## Data Availability

The original contributions presented in the study are included in the article/Supplementary Material, further inquiries can be directed to the corresponding authors.

## Abbreviations

CLI, Command-Line Interface; FDR, False Discovery Rate; UI, User Interface; GUI, Graphical User Interface; RMSD, Root Mean Square Deviation; TMscore, Template Modeling Score; QMEANDisCo, QMEAN Distance Constraints; NCBI, National Center for Biotechnology Information; BLAST, Basic Local Alignment Search Tool; LTS, Long-Term Support.
