# Supplementary material for: Prostruc: an open-source tool for 3D structure prediction using homology modeling
Source: Front Chem. 2024 Nov 29;12:1509407. doi: 10.3389/fchem.2024.1509407 (PMC11664737; doi:10.3389/fchem.2024.1509407)
Supplement: Supplementary file 1 [file DataSheet1.PDF]

Prostruc User Manual

1. Introduction

Prostruc is a comprehensive homology modeling tool designed to assist researchers in predicting protein structures based on known templates. Built with flexibility and accessibility in mind, Prostruc is ideal for bioinformaticians, structural biologists, and researchers who require accurate and customizable 3D protein models.

This manual provides guidance on installing, setting up, and using Prostruc effectively, covering everything from basic functionalities to advanced options.

Key Features

- sequence handling and batch processing
- Integration of Promod3 with ESMFold for deep learning-based structure prediction
- Modular refinement using ProMod3
- Preliminary structure validation Using TM score and QMean score

3. Getting Started

This section introduces Prostruc's interface and guides you through setting up your first project.

1. Launch Prostruc: Open the program from your applications menu or run the main script.
2. User Interface Overview:

1. Job Submission

Type a job name:

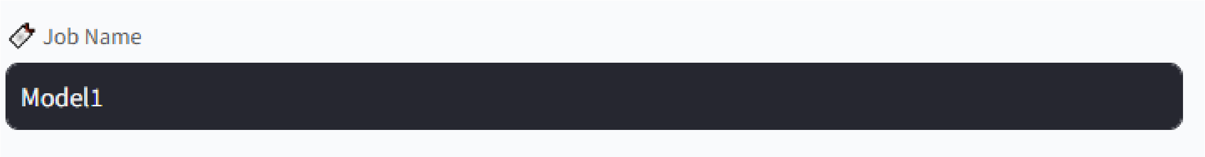

2. Input Data:

Protein amino acid sequence

The amino acid sequence of the target protein can be submitted either as plain text, or in FASTA format.

Example of plain text sequence:

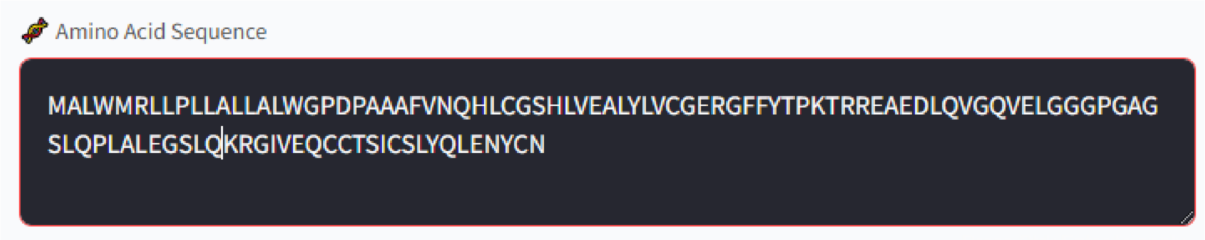

Example of FASTA sequence file:

```
>sp|P01308|INS_HUMAN Insulin OS=Homo sapiens OX=9606 GN=INS PE=1 SV=1
MALWMRLLPLLALLALWGPDPAAAFVNQHLCGSHLVEALYLVCGERGFFYTPKTRREAED
LQVGQVELGGGPGAGSLQPLALEGSLQKRGIVEQCCTSICSLYQLENYCN
```

Uploading FASTA File:

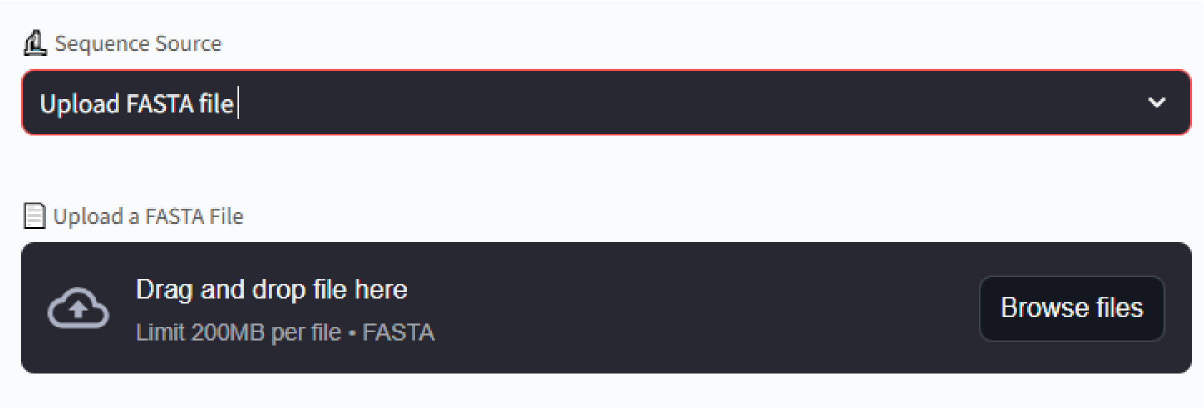

Provide email address to receive the results:

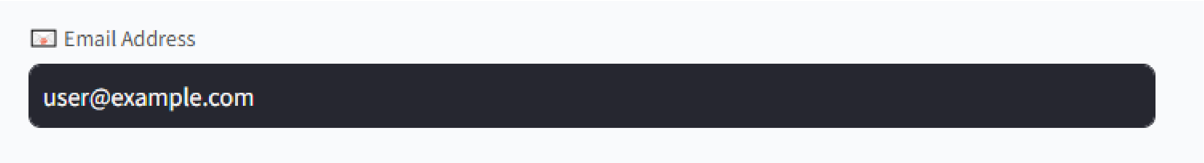

Click on submit job:

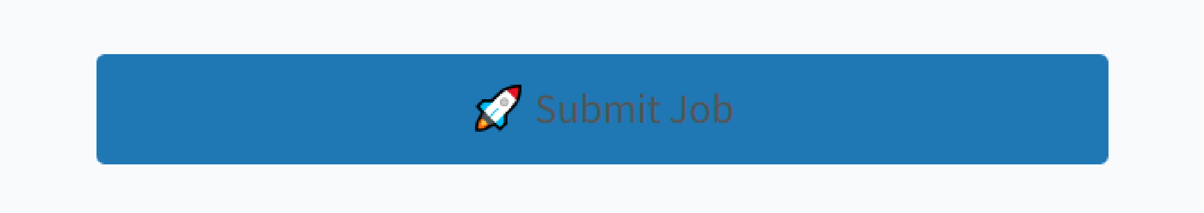

3. After submitting a job:

Check your email for job details:

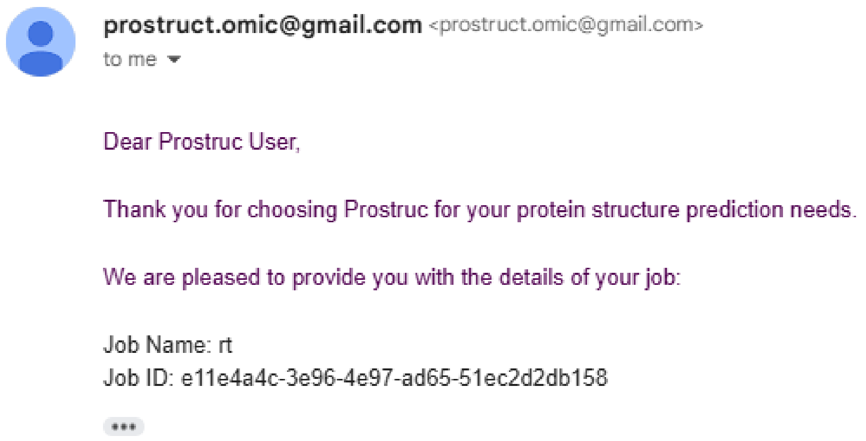

Copy them and navigate back to the prostruc web server, click on Job status

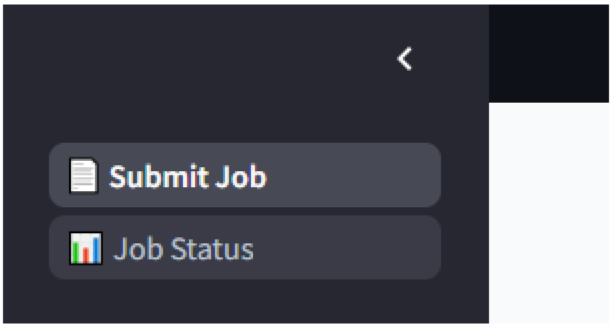

Enter the job details and click on check status

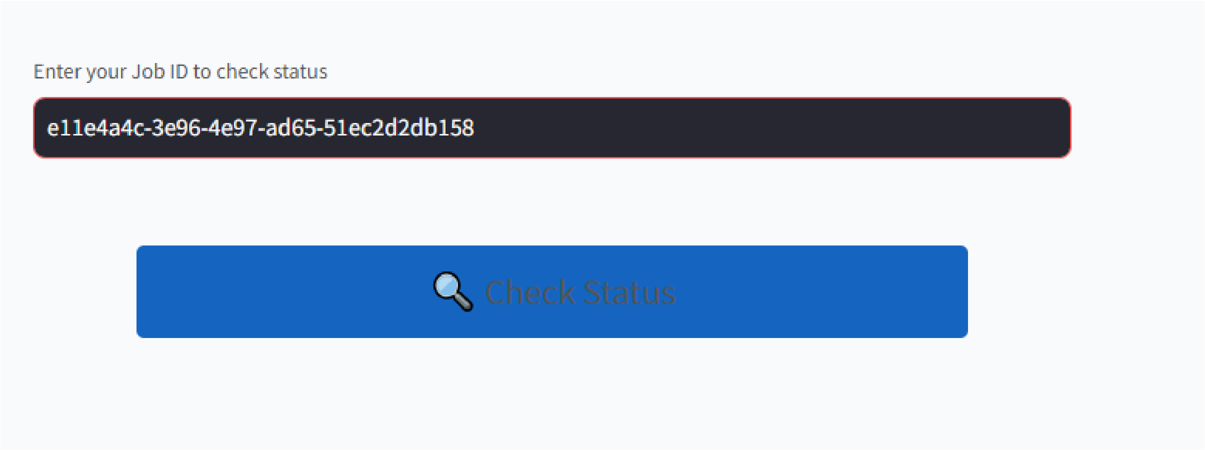

4. On completion:

Check your email for the results:

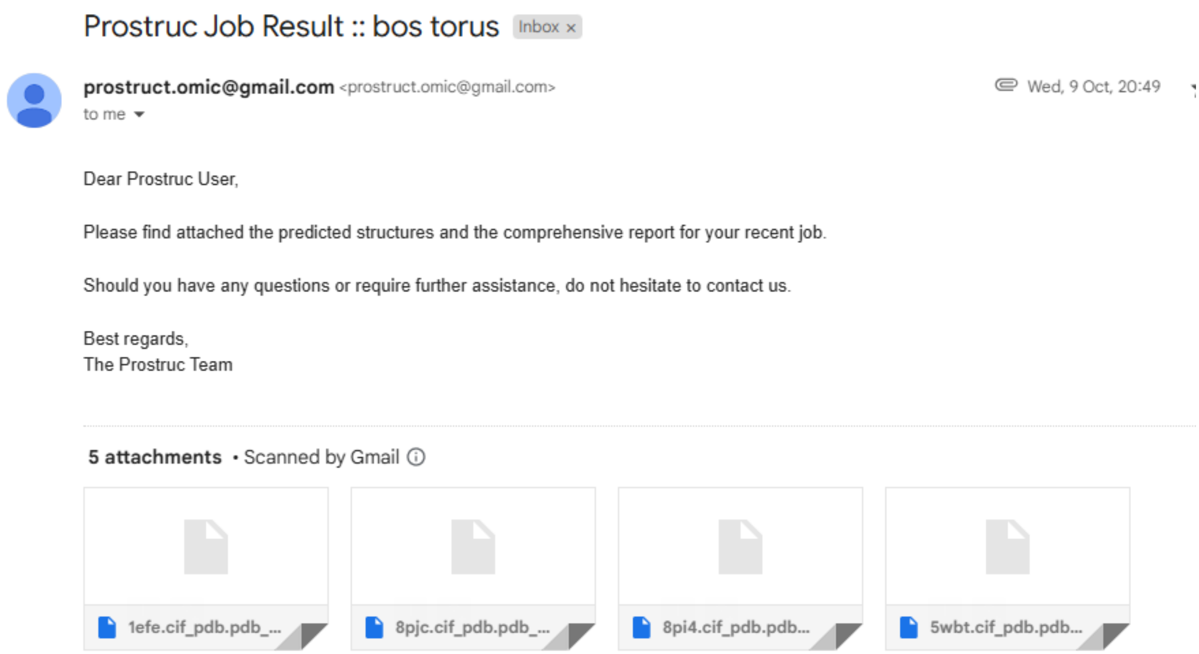

5. Results:

Download the PDB files and visualize them using any molecular visualization tool/software:

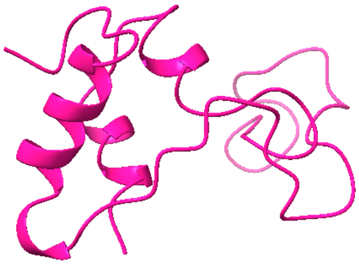

Fig 1. Predicted Model

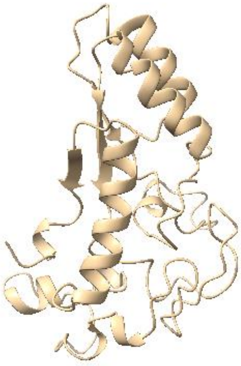

Fig 2.Predicted Model

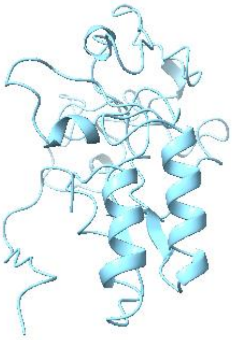

Fig 3.Predicted Model

3. Package Installation:

Installation

PROSTRUC is freely available as an open source package on the Windows platforms without the need for any license key or authorization.

Package Requirements

To ensure correct functioning of Prostruc, your machine needs to be actively connected to the internet and have a Python version 3.6 or later installed locally. You can download python via https://www.python.org/downloads/. If you already have python on your local machine, you can check your python version by using the following command:

```
python --version
```

Additionally, you must ensure that Docker is installed and actively running in the background. You can download docker at https://docs.docker.com/desktop/install/windows-install/. To verify Docker installation and status, use the following command:

```
docker --version
```

Alternatively, the Prostruc package can be installed using pip by running the following command:

```
pip install prostruc
```

NOTE: The user must log on as a Computer Administrator for the above features to effectively function (The first user is usually an administrator by default)

Getting started

Once Prostruc is successfully installed, please refer to the documentation https://github.com/omicscodeathon/prostruct/tree/main/scripts/prostruc#readme for a list of basic commands and required arguments. An example input script can also be found in the documentation section provided. To run the tool, use the following format:

Prostruc --sequence“ (Protein sequence)” --job_name “(enter_job_name)” --email”(enter_user_email)”.

For example:

```
python prostruc.py --sequence ″PROTEINSEQUENCE″ --job_name ″insulin_prediction″ --email ″user@example.com″
```

The current version works freely on windows provided the above package requirements have been met. Please also check periodically at the **patches** file in https://github.com/omicscodeathon/prostruct for hotfixes to the current version.
